# Exploring eHealth Ethics and Multi-Morbidity: Protocol for an Interview and Focus Group Study of Patient and Health Care Provider Views and Experiences of Using Digital Media for Health Purposes

**DOI:** 10.2196/resprot.2732

**Published:** 2013-10-17

**Authors:** Anne Townsend, Paul Adam, Linda C Li, Michael McDonald, Catherine L Backman

**Affiliations:** ^1^Milan Ilich Arthritis Research Center of CanadaRichmond, BCCanada; ^2^University of British ColumbiaDepartment of Occupational Science and Occupational TherapyVancouver, BCCanada; ^3^Mary Pack Arthritis Program, Vancouver Coastal Health AuthorityVancouver, BCCanada; ^4^University of British ColumbiaDepartment of Physical TherapyVancouver, BCCanada; ^5^W. Maurice Young Center for Applied EthicsSchool of Population and Public HealthUniversity of British ColumbiaVancouver, BCCanada

**Keywords:** ethics, eHealth, arthritis, multi-morbidity, patient/doctor engagement, self-management, patient role, Internet health, health tools, decision-aids

## Abstract

**Background:**

eHealth is a broad term referring to the application of information and communication technologies in the health sector, ranging from health records to medical consultations (telemedicine) and multiple forms of health education, support, and tools. By providing increased and anytime access to information, opportunities to exchange experiences with others, and self-management support, eHealth has been heralded as transformational. It has the potential to accelerate the shift from traditional "passive patient" to an informed, engaged, and empowered "patient as partner," equipped to take part in shared decision-making, and take personal responsibility for self-managing their illness.

**Objective:**

The objective of our study is to examine how people with chronic illness use eHealth in their daily lives, how it affects patient-provider relationships, and the ethical and practical ramifications for patients, providers, and service delivery.

**Methods:**

This two-phase qualitative study is ongoing. We will purposively sample 60-70 participants in British Columbia, Canada. To be eligible, patient participants have to have arthritis and at least one other chronic health condition; health care providers (HCPs) need a caseload of patients with multi-morbidity (>25%). To date we have recruited 36 participants (18 patients, 18 HCPs). The participants attended 7 focus groups (FGs), 4 with patients and 3 with rehabilitation professionals and physicians. We interviewed 4 HCPs who were unable to attend a FG. In phase 2, we will build on FG findings and conduct 20-24 interviews with equal numbers of patients and HCPs (rehabilitation professionals and physicians). As in the FGs conducted in phase I, the interviews will use a semistructured, but flexible, discussion guide. All discussions are being audiotaped and transcribed verbatim. Constant comparisons and a narrative approach guides the analyses. A relational ethics conceptual lens is being applied to the data to identify emergent ethical issues.

**Results:**

This study explores ethical issues in eHealth. Our goal is to identify the role of eHealth in the lives of people with multiple chronic health conditions and to explore how eHealth impacts the patient role, self-managing, and the patient-HCP relationship. The ethical lens facilitates a systematic critical analysis of emergent ethical issues for further investigation and pinpoints areas of practice that require interventions as eHealth develops and use increases both within and outside of the clinical setting.

**Conclusions:**

The potential benefits and burdens of eHealth need to be identified before an ethical framework can be devised.

## Introduction

### Background

eHealth can potentially transform how people live with and manage chronic illness. eHealth is a broad term referring to the application of information and communication technologies in the health sector, ranging from electronic health records to medical consultations (telemedicine) and multiple forms of patient information [1]. We limit eHealth in this study to the technologies used by patients to gather health information and support self-management, specifically, Internet use, decision-making tools, and monitoring systems [2]. “eHealth is an emerging field in the intersection of medical informatics, public health and business, referring to health services and information delivered or enhanced through the Internet and related technologies. In a broader sense, the term characterizes…a commitment for networked, global thinking, to improve health care…by using information and communication technology” [3]. The Canadian government has invested in this area since 1977 “eHealth is an essential element of health care renewal…Health Canada’s priorities and efforts have focused on addressing policy issues and challenges in mainstreaming eHealth services within Canada’s health care system and with measuring progress with the deployment and investment of these services” [1].

In 2009, 80% of 30,000 Canadian households and 73% of rural households in Canada used the Internet for personal reasons, while households in British Columbia reported the highest rates (85%). Of the Canadians who used the Internet, 70% used it to “search for medical or health related information,” up from 59% in 2007 [4].  A 2010 Pew survey in the United States showed that 74% of 3001 adults over the age of 18 used the Internet, of whom 80% (2065) sought health information [5]. The survey also showed that women are more likely to search for specific diseases and other medical problems, for themselves and others, reflecting their traditional role in family health. A lower percentage of people with chronic illness (n=1488) sought online health information than those who reported a recent experience of an acute episode (n=982) [5]. Fifty-three percent of adults with chronic conditions reported seeking health information online, compared to 62% of adults reporting no chronic conditions. Overall there was high motivation, especially among people living with chronic conditions, to connect with each other on the Internet. These figures suggest a lack of Internet access, rather than a lack of interest in health, as the primary reason for the gap. Those who accessed health information identified positive impacts such as gaining support for self-management and advice about negotiating pathways through care, learning from peers, gaining emotional support, and acquiring advice about treatment options [5]. Fox [5] suggests that this Internet access gap creates a gap in health information for people with chronic illness [5].

### Evidence of eHealth Influence

Given the potential role of eHealth in health care delivery, little evidence is available about how it is influencing patient-HCP relationships. A review of eHealth by Dedding et al [6] and HCP consultation identified five broad areas of impact. Positive impacts include (1) providing a replacement for face-to-face consultations, (2) supplementing existing relationships and forms of care, and (3) creating favorable circumstances for strengthening patient participation. On the other hand, eHealth may disturb the patient-HCP relationship (eg, some providers may feel threatened by patient knowledge and empowerment). Also, it demands more intense and frequent patient participation. Dedding concluded that experiences of patients are diverse, contradictory, and complex and that more research is required. Some evidence reveals a steep increase in patients who take health information found on the Internet into consultations [7,8]. Gauld's telephone survey [7] was based on a nonrepresentative sample of 406 Internet health users in Australia and New Zealand. He found patients increasingly use consultations to understand and confirm their Internet-acquired health information, 52% of Internet users had sought Internet health information alongside consulting their doctors, 40% consulted the Internet prior to their medical meeting, and 50% discussed health information they had found on the Internet with their practitioner. Of these Internet users (n=203), 15% believed their practitioner felt uncomfortable with this, 46% affirmed that it improved their relationship with their practitioner, and over 80% felt that it enhanced their understanding of the Internet-acquired information and treatment plan.

Another major form of Internet health use is found in peer-to-peer support or online forums where people share health concerns, experiences, information, and offer emotional and decision support [9] at all stages of chronic illness. A systematic review of online peer-to-peer support groups failed to show any benefits or harms to participants [10]. Another review of 47 studies concluded that while virtual communities have the capacity to empower consumers and improve service delivery, there is insufficient evidence regarding their effect on health outcomes [11]. The 2010 Pew Internet national survey of 3,000 respondents in the United States noted peer-to-peer help among people living with chronic conditions as a highly significant finding; 23% of Internet users living with a chronic condition reported going online to find others with similar health issues [5]. People can communicate with others in real time, remain anonymous, control the amount of personal information given, and benefit from the empathy of others who understand their fears [12], but there are potential problems such as the quality and trustworthiness of information shared [2]. For example, the stories of others may induce anxieties, offer misleading viewpoints, and inform decisions based on dubious information [13].

Self-management applications such as electronic devices for self-monitoring chronic conditions (eg, diabetes and asthma) have shown positive clinical effects in systematic reviews, but more evidence is called for [14]. A longitudinal study examined factors that encouraged use of a Web-based resource for monitoring diabetes, positive aspects included gaining feedback from an HCP and being encouraged to actively self-manage, while drawbacks included poor user friendliness [15]. Evidence shows that monitoring may improve patient awareness and adherence to treatments [16,17]. However, while self-monitoring systems are typically regarded as empowering for patients, they may also induce feelings of detachment and lead some patients to become more passive [18]. So, while technically effective, there may be negative impacts [19]. Quantitative research that examined self-monitoring in Type II diabetes found that monitoring could cause anxiety and depression and have a negative impact on quality of life [20]. Likewise, a review on self-monitoring blood pressure indicated it could be harmful to quality of life and encourages people to independently modify their treatment regimens. [21].

Decision aids are designed to inform and support individuals in making health and treatment decisions relevant to them and their illness condition through presenting information, options, and outcomes [22]. Increasingly electronic and interactive, they are regarded as important components of eHealth [2]. One review of 55 clinical trials involving 51 different decision aids showed multiple positive benefits such as encouraging patients to be more actively involved with treatment decisions without causing anxiety [22]. Criticisms have also been aimed at decision aids, in particular that they can be insensitive to the needs of individual patients [23]; this is especially relevant to those with multi-morbidities and complex treatment regimens.

There is some evidence about physician responses to eHealth. Jacobson’s review [24] shows that there is resistance from physicians regarding online health information [25,26]. Oncologists displayed skepticism relating to the nature and quality of Internet information and the potential for inducing anxiety or false hope in patients [27]. A focus group study in Canada with 48 family physicians showed the physicians had concerns regarding patients being misinformed by online information, which could cause confusion, distress, and inappropriate self-treatment [28]. Another concern was the time it took to explain Internet information [28,29], which could cause frustrations and tensions in patient-doctor relations [30]. Other studies show significant variation in how specialists respond to patients. There is some evidence that a very small percentage of professionals recommend that their patients use the Internet or refer to the Internet during in-person consultations [31], while some physicians have described the Internet as having a positive effect [32]. In one cross-sectional survey of a nationally representative sample of United States physicians (1050 respondents; response rate 53%), 75% of physicians considered the increase in health information on the Internet to be “a good or very-good thing.” Thirty-eight percent (n=399) of physicians believed that bringing Internet information to the consultation was either positive or neutral [29]. A study of male cancer patients suggested that the Internet strengthened the patient-physician relationship, as it prompted discussions about online information [27]. These changes in the patient role have implications for physician training. Bos [33] undertook a review of patient empowerment in 2012 and concluded that HCPs will need training to deal with knowledgeable patients and emerging ethical issues in eHealth [33].

Given the potential of eHealth to transform illness experience and the delivery of care, it may be particularly significant for people with multi-morbidity and associated wide-ranging information and complex self-management needs [34]. The explosion in online health information and support groups, electronic tools, and decision aids aligns with rising trends in self-management of chronic conditions and healthy consumer self-care [35]. Self-management programs for arthritis, diabetes, and other chronic diseases encourage patients to take on an active role and promote individual responsibility in illness management [36,37], and there is evidence to show that self-management programs are associated with positive outcomes [37]. Despite there being a lack of robust evidence [38], eHealth is considered a key tool to extend the reach of self-management programs with the capacity to more successfully align initiatives with patient needs, support and educate individuals to more actively participate in their health management [39], encourage patient-provider partnerships in health care [40], and “empower” people to maintain control of their illness and lives [2]. There is optimism about eHealth’s potential to improve health care processes and patient outcomes [41]. The informed patient uses the Internet to seek second opinions, understand symptoms, get support, gain clarification of HCPs’ advice, and devise questions for future consultations [42]. Policy documents from Health Canada’s Office of Health and the Information Highway and the Department of Health in the United Kingdom also suggest that more informed patients would be empowered and equipped to better manage their health [24,43].

### Aim of the Study

The main aim of this study is to provide a systematic ethical analysis of emerging issues in eHealth regarding its role and impact on chronic illness experience and management. Specific objectives are to:(1) identify, understand, and compare how men and women with multi-morbidity (arthritis plus one or more chronic conditions) use eHealth, both broadly and with particular attention to their gathering of health information, decision-making, and self-management, (2)investigate how eHealth impacts patient-provider relationships, and (3) identify and address ethical ramifications of eHealth for patients, providers, and health service delivery.

Our research will contribute to a better understanding of the role of eHealth in managing multi-morbidity from patient and HCP perspectives. eHealth has been identified as a catalyst for positive and sweeping improvements, but only provisional empirical evidence on how consumers engage with eHealth and conflicting evidence about its impact on patient-provider relationships exists. Focusing on experiences of multi-morbidity, to illuminate issues of use and need, this study will identify and analyze emerging ethical issues of eHealth domains for self-management and patient-provider relationships. Technologies might not deliver their potential if we do not gain a comprehensive appreciation of benefits and harms. For example, the study may illuminate how eHealth affects empowerment, autonomy, and equity on individual, interactional, and systems levels in different practice contexts. Distinguishing the salient themes will help to guide further research and begin to identify areas for both patient and HCP support and the implications for decision-making and the patient-HCP relationship.

### Ethical Framework and Study Design

A relational ethics approach is suitable to identify and analyze the potential benefits and harms of a range of eHealth sources and address how far they enhance patient-provider interactions and support genuine decision-making. Moral reasoning can provide a framework to address ethical considerations in eHealth. While drawing on the traditional bioethics principles of autonomy, beneficence, and justice, we adopt a relational approach to understanding the role and ramifications of eHealth. Relational ethics emphasizes context. For example, the traditional conceptualization of autonomy offers an individualistic model of human agency, emphasizing independence and individual competence [44]. Relational autonomy prioritizes interdependence, relationships, social, and structural factors that facilitate or constrain meaningful self-direction [44]. As such, relational ethics is equipped to tackle a range of ethically complex situations arising in eHealth.

Empowerment is claimed as a major benefit of eHealth, but the process of becoming empowered and what this means to patients and HCPs is not well understood. For example, an overemphasis on empowerment may be harmful to patients. If there is a focus on individual patient’s ability and desire to be empowered, people may feel responsible for underachievement of outcomes to control a disease or symptoms. This may be especially challenging for those managing multiple diseases with conflicting recommendations and multiple medications for different health problems [45]. A review of eHealth by Dedding et al [6] raises questions about the redistribution of tasks and responsibilities to patients as consumers, and how far this becomes an added burden in daily life [6]. Being self-sufficient and informed may place unrealistic and burdensome expectations on the sickest and exacerbate disadvantage. Some people may resist the new patient role, not have the resources, or lack an HCP who supports their developing empowerment [46,47]. This critical stance mirrors research in sociology about the work of chronic illness and self-management [48], and an ethics perspective on “patient work” [49]. Relational autonomy emphasizes the complex webs of personal and institutional relationships that facilitate real choice and offer ways of respecting another’s autonomy. In this way, it addresses the daily life context of patients and the patient-HCP encounter and engages critically with the concept of empowerment and the context in which it emerges and is supported [6].

eHealth poses situations of fundamental moral uncertainty and conflicts between competing values and responsibilities in patient-provider relationships [6]. For example, some providers may find it difficult to relinquish traditional roles and a challenge to gain skills in new partnership-based roles. We do not know how issues of trust and agency are impacted by eHealth. There may be tensions between the development of trust and patient empowerment, which is seen as a major benefit of eHealth as well as prioritized as a policy goal. Interpersonal trust has traditionally been based on imbalance in the medical encounter, the vulnerability of the patient and the specialized knowledge of the HCP [50]. Given the potential shift in the patient-provider relationship, forming a partnership of trust requires knowledge and skills to encourage genuine partnership. For example, traditionally HCPs have been the gatekeepers to health information, but Internet information is an integral part of patient experience. The trust relationship and clinicians’ fiduciary responsibility to be beneficent and avoid harm extends to respecting, recognizing, and supporting the patient’s perspective and listening to patients as partners. Harm may ensue if patients’ skills, experience, and knowledge are devalued [46], which is inconsistent with providers’ obligations of fidelity and compassion [51], and their role in providing meaningful support. A relational approach to autonomy will examine the perspectives of both consumers and HCPs in the context of this cultural shift in care and address a range of issues including mutual trust, shared decision-making, and responsibility.

Individuals with poorer health status may also have less access to eHealth tools, so the expansion of eHealth could *exacerbate* health disparities. Even if there is equal access to eHealth resources, its potential benefits may remain beyond reach for some individuals/groups. Access alone, if not accompanied by services, support, and resources designed to reach and appeal to diverse populations, will not automatically improve an individual’s eHealth use, or their health outcomes. The concept meaningful access, recognizes that in addition to physical access to eHealth, individuals need the skills and resources to use eHealth tools on a sustained basis. Issues of equity need to be considered regarding disparity in access to skills, education, and opportunities to develop them. Some people live complex lives compromised by illness and face adverse social conditions and personal circumstances that may place constraints on what they can accomplish via eHealth. Multiple disadvantages and vulnerabilities may compound illness and how illness is faced. In this way, structural, personal, and cultural factors may compromise or support optimum use of eHealth. Equity issues also include access to suitable equipment, Internet connections, opportunities for skill development, ongoing technical support, and web content that is appropriate for diverse users. Meaningful access also requires appropriate daily life situations, HCP, and health services support. A relational ethics approach will address issues of access in context, or meaningful access to eHealth and its ramifications.

## Methods

### Two-Phase Study

In this two-phase study, we use a qualitative approach, suitable to investigate process, social settings, human behavior, and examine how individuals make sense of their world. In this study we are guided by grounded theory [52] and narrative [53]. We apply a “social constructionist version” of grounded theory that aims to gain an interpretive understanding of social phenomena [52], emphasizing flexibility, replacing the more formulaic approach of original grounded theory [52]. We attempt to construct theory from the data, and will draft an explanatory framework for future study. We also draw on a narrative approach to hear people’s storied accounts of their lives and experiences, how they build coherence, and link action with a moral purpose. This helps us recognize the moral themes of accounts. This fits with our focus on the ethics of health care and our overarching framework of relational ethics. In phase one of this study we conducted focus groups to gain insight into a range of perspectives and experiences of eHealth from a range of HCPs and patients. In part, this was a pragmatic choice, because it gave us the opportunity to relatively easily collect data from several perspectives simultaneously. Methodologically, we also wanted to encourage group discussion so that we could explore what people thought, how they thought, and why they thought that way [54]. We are in the process of identifying emergent themes that we will use as a basis for the phase-two interview topic guide for patients and HCPs for in-depth investigation.

### Rationale for Participant Sample

**Figure 1:**
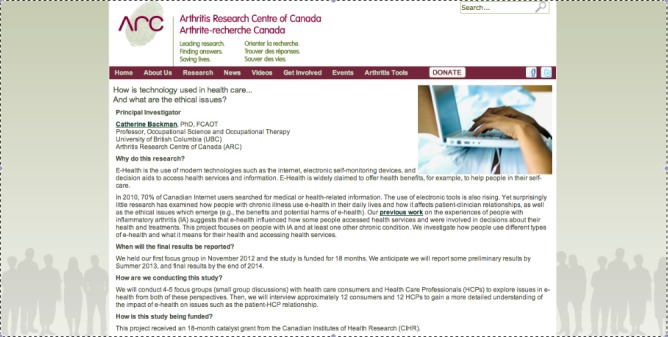
Arthritis Research Center of Canada screenshot of Web page concerning how technology is used in health care research.

We selected people with arthritis and co-conditions for this study for two reasons-pragmatism and prevalence. We have an established, excellent working relationship with the Arthritis Research Center of Canada. Building on our knowledge base and research relationships enhanced recruitment and study feasibility. Figure 1 shows a screenshot from the Research Centre’s Web page. Arthritis is a highly prevalent and serious chronic condition, the leading cause of pain and disability in Canada [55], hampering meaningful activity across life domains. The Canadian Community Health Survey (CCHS), (124,844 respondents, response rate 76%) based on 2007-2008 data, estimated that more than 4.2 million Canadians 15 years and older (16% of the population) had arthritis [55]. The coexistence of other chronic conditions with arthritis was reported as common by the Public Health Agency of Canada, based on the CCHS 2007-2008 data; both men and women frequently reported back problems (42.5%, 41.6%, respectively), high blood pressure (34.7%, 39.1%), heart disease (14.7%, 12.3%), diabetes (14.4%, 13.3%), and mood or anxiety disorders (13.3%, 19.5%) [55]. These coexisting conditions pose problems for individuals, populations, and HCPs, complicating effective treatment and disease management [56]. Multi-morbidity (the presence of two or more co-occurring chronic conditions) becomes more common as populations age, and will rise [57]. Research until very recently has however, tended to focus on single conditions.

Multi-morbidity is increasingly common. A 2003 Canadian study concluded that patients with multi-morbidity seen in family practice were the rule rather than the exception [34]; the province of British Columbia reported that in 2005/2006, 1.3 million patients had 1-3 confirmed chronic conditions and over 92,000 had 4 or more confirmed chronic conditions [57]. Multi-morbidity is associated with high burdens of care and cost [58]. Despite this, our knowledge and understanding of the impact of multi-morbidity for patients and HCPs is poor [59]. Furthermore, despite the explosion of eHealth, we are aware of very little research, if any, on the ethical implications of its role, the impact on self-management, and the patient-HCP relationship in multi-morbidity. Because eHealth is a vast resource for both consumers and HCPs, it is vital to identify its potential benefits and harms, perhaps particularly salient for those who have multi-morbidity and their HCPs who deal with more information and more complex decisions. By using our existing relationships in the arthritis community, we continue to efficiently recruit patients and HCPs with a focus on a common chronic condition as a unifying thread, and concomitantly explore the complexity of multi-morbidity.

### Participants and Procedures

We aim to recruit a total number of approximately 60-70 participants for FG discussions and in-depth interviews. This sample comprises an equal number of patients and HCPs. All participants will be adults with self-reported diagnosis of arthritis and one other condition, and use online health resources. This number was considered appropriate on methodological and practical grounds. It is feasible given the time of the research over 18 months; it allows comparisons between groups to identify patterns and range of experiences. Also, because of the amount of data generated in qualitative inquiry, this number still allows for in-depth analysis of data. A purposive sample will be recruited using online groups and listservs, newsletters, websites, posters in clinical settings and offices, word-of-mouth, and community advertising. The inclusion criteria for HCPs are a minimum of 2 years experience working with people with chronic conditions, and who report at least 25% of their caseload has more than one chronic condition. The inclusion criteria for patients are a diagnosis of arthritis (eg, rheumatoid arthritis, ankylosing spondylitis) and at least one additional chronic condition of any duration. For practical reasons, participants live in British Columbia and are able to converse in English. We aim for variation in socio-demographic characteristics including age, gender, geographical location, years of experience and professions (HCP), or disease duration and education (patients). Family members/caregivers of patients will be able to participate in the study.

We planned 8 FGs of 4-8 participants, with patients and HCPs in separate groups–at least four groups with HCPs (including family physicians, specialist physicians, nurses, occupational and physical therapists) and the remainder with patients. The FGs have been and the interviews will be facilitated/conducted by members of the research team experienced in qualitative research (AT and PA). Group discussions lasted approximately 2 hours, plus a short break. For the in-depth interviews we plan to recruit approximately 12 patients and 12 HCPs in order to gain perspectives of both groups (24 interviews in total). Based on our previous qualitative studies on living with chronic conditions, we expect interviews to last approximately 90 minutes. All interview participants will receive a telephone follow-up call of 20-30 minutes that will serve to verify, clarify, and expand on issues discussed.

The audiotaped FGs began by exploring topics, which will be refined and explored further in the interviews. Content focused on forms of eHealth and their impact for self-management and patient-provider relationships, and were organized into three sections. First, broad questions were asked to explore how participants used or viewed eHealth, what kinds of information they needed and preferred, and what sort of decisions they considered making based on eHealth information. Examples of eHealth formats mentioned were: (1) peer-to-peer online support groups, (2) Internet use in general, (3) decision aids, and (4) self-management monitoring devices and applications. The FG guide was arranged around four key areas (1) Devices and types of eHealth used, (2) Details about reasons for use, (3) How eHealth use influenced actions taken including interactions with HCPs, and (4) The benefits and harms/drawbacks of eHealth. (This fourth section probed about ethical issues of eHealth both explicitly and implicitly). Participants were encouraged to compare and contrast their use and views of different types of information and how it related to their experiences. To encourage maximum engagement from all participants, sessions were as relaxed as possible, with refreshments and a comfortable setting (eg, seated in a circle). The facilitator encouraged each participant to contribute to all sections of the topic guide, and to talk to each other and not address themselves solely to the facilitator. In this way focused conversation was fostered rather than questions/answers format. A flip chart was used to note key points and for more focused probing and elaboration. The level of discussion of each issue varied between and within groups. We anticipated that the FG would be unlikely to fully explore all the questions; consequently they were used to generate preliminary data and to identify the most salient topics and findings to inform an interview guide for more in-depth exploration with individuals (see Multimedia Appendix 1 for key FG questions).

### Analysis

An iterative, thematic approach using constant comparative methods is being applied to the data. The audiotaped FG discussion transcripts have been, and the interviews will be, checked against recordings for accuracy and anonymized. We will agree on conceptualizations of relational ethics as an overarching analytic framework. The FG analysis draws on aspects of grounded theory-simultaneous collection and analysis of data, two-step data coding process, constant comparative methods, and memo writing. Three researchers read and annotated a sample of transcripts independently and after discussion agreed on a broad initial coding framework, which will be applied to all transcripts using QSR NVivo7 software. This allows data storage, organization, and constant comparisons within and between transcripts. We will modify and add codes in the light of fresh transcripts and repeated readings. We applied initial themes to all transcripts (eg, building trust). After further analysis higher-level themes will emerge (eg, informed trust, trust wariness). We are identifying both a priori and emerging themes. A summary analysis will provide themes for the interview guide. Interview analysis similarly will follow grounded theory as above. The data generated will be more in-depth and also allow a narrative analysis. We will look for three core narratives-stability, progressive, and regressive [53]. In this way, the analysis is drawn to process, morally informed actions, and decision-making. Applying a relational ethics lens to the dataset, emerging themes include issues related to autonomous decision-making, building trust, hampering trust, building partnerships, taking control, giving control, and sharing responsibility. The ethical analysis will be interpreted in the context of the current literature and e-sources. It will also guide the development of a future more extensive investigation of greater scope (of the most salient eHealth ethical concerns, with a range of people) with patients, clinicians, and caregivers, to assess the transferability of the findings about access, benefits/burdens of eHealth, and communication in consultations.

### Ethics Approval

We obtained ethics approval for the research from the University of British Columbia Behavioral Research Ethics Board and Vancouver Coastal Health Research Institute. Participants in this study will be provided with a detailed information sheet describing the study and sufficient information to make an informed decision about participation before they give written consent. Participants will be informed they can withdraw from the study at any time.

## Results

This paper presents a protocol of a study in progress, and results are not yet known. The current project status is as follows–Between November 2012 and June 2013 we recruited 36 participants (18 patients, 18 HCPs). The participants attended 7 FGs, 4 with patients and 3 with rehabilitation professionals and physicians. We interviewed 4 HCPs who were unable to attend an FG. Preliminary analysis revealed that patients and HCPs expressed similar views about eHealth, though examples, emphasis, and priorities varied. Analysis is ongoing and findings from the FGs are anticipated by October 2013. Building on the main themes to emerge from this FG phase of the study, we will create topic guides to conduct interviews between October and November 2013. Results from the interviews are anticipated by March 2014.

## Discussion

### Qualitative Studies

Like many qualitative studies, we will gain retrospective accounts of participant experiences. Because we are not asking participants to relay objective facts, but subjective experiences most significant to them, they are likely to recount potent factors, episodes, and processes associated with how they experienced eHealth, multi-morbidity, and clinical encounters, which is our main interest. A well-designed qualitative study is an efficient method for capturing a wide range of experience from the individual’s perspective, while minimizing the chance of missing salient factors due to recall bias. Recruiting from British Columbia alone is a potential limitation in terms of generalizability. However, we can estimate how far our findings will be transferable to other settings. Our sampling approach is a practical solution to reach people with arthritis and at least one other condition and HCPs.

### Chronic Illness and eHealth

Surprisingly little research has examined how people with chronic illness use eHealth in their everyday lives, how it affects patient-HCP relationships, or its ethical ramifications for patients, providers, and service delivery. This study examines these issues, drawing on traditional bioethics principles of autonomy, beneficence, and justice within a more recent framework of relational ethics. Internet use is enormously diverse with numerous formats of factual sites, which can be accessed at any time, encouraging rising health consumerism [2]. People increasingly use the Internet to proactively manage their health [30,32]. There is some evidence to show the Internet influences decision-making. In a US survey drawing on a sample of 60,000 households, responses were analyzed from 4764 individuals 21 years and older who self-reported as Internet users. Forty-three point seven percent reported more than one chronic condition, about half of whom indicated Internet use improved their understanding of their chronic conditions and treatments for their chronic condition or other symptoms and treatments. “The percentage indicating effects on decisions about health or health care or on use of the health care system ranged from 7% to 32%” [60]. There are concerns about the quality and quantity of health information on the Internet [2] and we have limited knowledge of how consumers engage with eHealth [2]. Gauld [7] reported that 90% of respondents believed that health information obtained over the Internet was trustworthy, yet only 35% consistently checked the credentials of the information source. Research to date has only begun to tap into the frequency of Internet use and patient-provider communication; more in-depth study of the influence of this information is necessary. One example for further study is to assess whether or not integrating Internet data into the patient-provider consultation lengthens the visit or affects attitudes, communication style, or intervention plans to better understand the role of eHealth information in patient engagement and empowerment. Given the enormous potential eHealth holds for transforming and enhancing health care delivery and self-management, we know relatively little about how eHealth impacts the patient-HCP relationship. We have insufficient evidence of the potential harms and benefits of Internet use for people's health. Gaining evidence as a precursor to introducing costly interventions based on enthusiasm rather than evidence is also an ethical undertaking. Although claims have been made about the potential transformative promise of eHealth for health service delivery [2], more patient-centerd care and complex changes to patient-HCP relationships [51], we have disarmingly little data on its impact in terms of potential benefits and harms and if access gaps equal a gap in health care and self-management support. This situation raises profound ethical issues, which we will examine.

## Multimedia Appendix 1

"Notice of decision" document. This contains the reviewer comments from the the Canadian Institutes of health Research (CIHR), who funded this project.

## Abbreviations

CCHS: Canadian Community Health Survey
FG: focus group
HCP: health care provider
