# Supplementary material for: Exploring eHealth Ethics and Multi-Morbidity: Protocol for an Interview and Focus Group Study of Patient and Health Care Provider Views and Experiences of Using Digital Media for Health Purposes
Source: JMIR Res Protoc. 2013 Oct 17;2(2):e38. doi: 10.2196/resprot.2732 (PMC3806546; doi:10.2196/resprot.2732)
Supplement: Supplementary file 1 [file resprot_v2i2e38_app1.pdf]

May/Mai 23, 2012
DCO150GP

Canadian Institutes of Health Research/Instituts de recherche en santé du Canada

Notice of Recommendation/Avis de recommandation

Application Number/Numéro de la demande: 274771
Committee Code/Code du comité: EPP

Applicants/Candidats: Dr. Catherine Louise BACKMAN

With/Avec: Mr. P. ADAM   Dr. A. TOWNSEND

Institution paid/ Établissement payé: University of British Columbia

Title/Titre: Exploring E-health ethics and multi-morbidity

Primary Inst./Inst. principal: Musculoskeletal Health and Arthritis
Other Related Inst./ Autres inst. connexes: Health Services and Policy Research

Competition /Concours: Catalyst Grant: Ethics
March/Mars 01, 2012

Number in competition/Nbre de demandes dans le concours: 10

Peer Review Committee Recommendation, for your information and use/ Recommandation du comité d'examen par les pairs, pour fins d'information et d'utilisation:

**Committee/Comité:**	Catalyst Grant: New Investigators and Mid-Career Investigators Transitioning into Ethics
**Application rank within the competition/ Rang de la demande dans le concours:**	5
**Percent Rank within the competition / Rang en pourcentage au sein du concours:**	50%
**Rated / Cote:**	3.80
**Recommended Term/ Durée recommandée:**	1 years/ans 6 months/mois
**Recommended average annual operating amount/ Montant annuel moyen recommandé pour le fonctionnement:**	$47,130
**Recommended equipment amount/ Montant recommandé pour les appareils:**	$0

This document is for information only.
An application rated below 3.50 is ineligible for CIHR funding. For applications rated 3.50 and above, please note that it is the application's rank within the peer review committee that determines whether it is funded, rather than its absolute rating. The final funding decision will be communicated in the Notice of Decision.

Document à titre d'information seulement.
Une demande cotée en dessous de 3,5 n'est pas admissible au financement des IRSC. En ce qui a trait aux demandes cotées 3,50 ou plus, veuillez noter que l'on détermine l'attribution des fonds en fonction du classement obtenu au sein du comité d'examen par les pairs plutôt qu'en fonction du classement absolu. La décision finale relative au financement sera communiquée dans l'Avis de décision.

June/Juin 08, 2012

DC0190GP

Canadian Institutes of Health Research / Instituts de recherche en santé du Canada

Notice of Decision / Avis de décision

Application Number/Numéro de la demande: 274771

Committee Code/Code du comité: EPP

Applicants/Candidats: Dr. Catherine Louise BACKMAN

With/Avec: Mr. P. ADAM   Dr. A. TOWNSEND

Institution paid/ Établissement payé: University of British Columbia

Title/Titre: Exploring E-health ethics and multi-morbidity

Primary Inst./ Inst. principal: Musculoskeletal Health and Arthritis

Other Related Inst./ Autres inst. connexes: Health Services and Policy Research

**Competition Outcome/Résultats du concours:**	Catalyst Grant: Ethics<br>March/Mars 01, 2012
**Number in competition/Nbre de demandes dans le concours:**	10
**Number approved/Nbre de demandes approuvées:**	5

**Decision on your application/ Décision sur votre demande:**	Approved
**Average annual amount/ Montant annuel moyen:**	$47,130
**Equipment amount/ Montant pour les appareils:**	$0
**Term/Durée:**	**1 yrs/ans 6 months/mois**

Peer Review Committee Recommendation, for your information and use/
Recommandation du comité d'examen par les pairs, pour fins d'information et d'utilisation:

**Committee/Comité:**	Catalyst Grant: New Investigators and Mid-Career Investigators Transitioning into Ethics
**Application rank within the competition/ Rang de la demande dans ce concours:**	5
**Percent Rank Within the Competition/ Rang en pourcentage au sein du concours:**	50%
**Rating/ Cote:**	3.80
**Recommended average annual amount/ Montant annuel moyen recommandé:**	$47,130
**Recommended equipment amount/ Montant recommandé pour les appareils:**	$0

*** Applications receiving a score of less than 3.5 on any evaluation criteria will not be considered for Funding. / Les demandes qui ont reçu une note inférieure à 3.5 pour n'importe quel des critères d'évaluation ne sont pas admissibles.

This document is for information only. Official payment is stated on the CIHR Authorization for Funding.
Document à titre d'information seulement. Le paiement officiel est indiqué sur l'autorisation de financement des IRSC.

CIHR IRSC

Institute of Aboriginal Peoples' Health
Institute of Aging
Institute of Cancer Research
Institute of Circulatory and Respiratory Health
Institute of Gender and Health
Institute of Genetics
Institute of Health Services and Policy Research
Institute of Human Development and Child and Youth Health
Institute of Infection and Immunity
Institute of Musculoskeletal Health and Arthritis
Institute of Neurosciences, Mental Health and Addiction
Institute of Nutrition, Metabolism and Diabetes
Institute of Population and Public Health

Institut de la santé des Autochtones
Institut du vieillissement
Institut du cancer
Institut de la santé circulatoire et respiratoire
Institut de la santé des femmes et des hommes
Institut de génétique
Institut des services et des politiques de la santé
Institut du développement et de la santé des enfants et des adolescents
Institut des maladies infectieuses et immunitaires
Institut de l'appareil locomoteur et de l'arthrite
Institut des neurosciences, de la santé mentale et des toxicomanies
Institut de la nutrition, du métabolisme et du diabète
Institut de la santé publique et des populations

June 8, 2012

Dr. Catherine Louise BACKMAN
The University of British Columbia
OccupationalScience &OccupationalTherapy
Room T325, third floor, Koerner Pavilion
2211 Wesbrook Mall
Vancouver, British Columbia V6T 2B5

Dear Dr. BACKMAN:

I am pleased to inform you that CIHR (Canadian Institutes of Health Research) has approved your recent application to the Catalyst Grant: Ethics entitled "Exploring E-health ethics and multi-morbidity". As you are receiving this letter through ResearchNet your Authorization for Funding will follow in the mail.

If you have not already received the review documents related to your proposal, please contact us. Should you have any questions about the review process, please address them directly to CIHR staff. Do not contact the officers or members of the peer review committee. As CIHR does not notify co-applicants of the decision, we ask that you inform those individuals involved, along with their research institutions (if different from your own), of the outcome of this application.

Congratulations on your success in this competition.

Gregory Huyer, Ph.D.
Deputy Director, Knowledge Creation Programs
Research and Knowledge Translation Portfolio

301317-201203EPP-EPP-274771-49294-RSRFA

Canadian Institutes of Health Research
Room 97, 160 Elgin Steet, Address locator: 4809A
Ottawa, (Ontario) K1A 0W9 Tel.: (613) 941-2672
Fax (613) 954-1800 **www.cihr-irsc.gc.ca**

Instituts de recherche en santé du Canada
Pièce 97, 160 rue Elgin, Indice de l'adresse: 4809A
Ottawa, (Ontario) K1A 0W9 Tél.: (613) 941-2672
Fax (613) 954-1800 **www.irsc-cihr.gc.ca**

Canada

CIHR IRSC

Institute of Aboriginal Peoples' Health

Institute of Aging

Institute of Cancer Research

Institute of Circulatory and Respiratory Health

Institute of Gender and Health

Institute of Genetics

Institute of Health Services and Policy Research

Institute of Human Development and Child and Youth Health

Institute of Infection and Immunity

Institute of Musculoskeletal Health and Arthritis

Institute of Neurosciences, Mental Health and Addiction

Institute of Nutrition, Metabolism and Diabetes

Institute of Population and Public Health

Institut de la santé des Autochtones

Institut du vieillissement

Institut du cancer

Institut de la santé circulatoire et respiratoire

Institut de la santé des femmes et des hommes

Institut de génétique

Institut des services et des politiques de la santé

Institut du développement et de la santé des enfants et des adolescents

Institut des maladies infectieuses et immunitaires

Institut de l'appareil locomoteur et de l'arthrite

Institut des neurosciences, de la santé mentale et des toxicomanies

Institut de la nutrition, du métabolisme et du diabète

Institut de la santé publique et des populations

June 8, 2012

Dr. Catherine Louise BACKMAN
The University of British Columbia
OccupationalScience &OccupationalTherapy
Room T325, third floor, Koerner Pavilion
2211 Wesbrook Mall
Vancouver, British Columbia V6T 2B5

Dear Dr. BACKMAN:

Congratulations on your success in the recent Canadian Institutes of Health Research funding competition. You should take great pride in your success, particularly in light of the very competitive nature of CIHR peer review.

As you know, peer review is the cornerstone of our research funding system. This process rests on the kind voluntarism of your colleagues at other institutions who generously gave their time to review your application.

The Canadian Institutes of Health Research is committed to building an innovative national health research enterprise. To this end we have undertaken the development of a renewed strategic plan for CIHR, our Health Research Roadmap which has required support from researchers, policy makers, the voluntary sector and the Canadian public. To meet CIHR goals, we must share our knowledge. That is why we encourage you to work with your institution to communicate to Canadians about the work you are doing. To simplify this process, we have developed guidelines on public communication which you can find on our website at http://www.cihr-irsc.gc.ca/e/30789.html.

Once again, congratulations and I wish you success in your research.

Yours sincerely,

Alain Beaudet, MD, Ph.D.
President

301319-201203EPP-EPP-274771-49294-CONGR

President

Canadian Institutes of Health Research
Room 97, 160 Elgin Steet, Address locator: 4809A
Ottawa, (Ontario) K1A 0W9 Tel.: (613) 941-2672
Fax (613) 954-1800 **www.cihr-irsc.gc.ca**

Président

Instituts de recherche en santé du Canada
Pièce 97, 160 rue Elgin, Indice de l'adresse: 4809A
Ottawa, (Ontario) K1A 0W9 Tél.: (613) 941-2672
Fax (613) 954-1800 **www.irsc-cihr.gc.ca**

Canada

Review Type/Type dévaluation: Committee Member 1/Membre de comité 1
Name of Applicant/Nom du chercheur: BACKMAN, Catherine Louise
Application No./Numéro de demande: 274771
Agency/Agence: CIHR/IRSC
Competition/Concours: 2012-03-01 Catalyst Grant: Ethics/Subvention catalyseur : éthique
Committee/Comité: Catalyst Grant: New Investigators and Mid-Career Investigators Transitioning into Ethics/Subvention catalyseur : Nouveaux chercheurs faisant la transition au domaine de l'éthique
Title/Titre: Exploring E-health ethics and multi-morbidity

Assessment/Évaluation:

Synopsis

The proposal addresses the increasingly important topic of how people with chronic conditions use e-health. The goal is to provide a systematic ethical analysis of emerging issues in e-health regarding its role and impact on chronic illness experience and management. The applicants limit e-health to the technologies used by the public and patients to gather health information and support self-management, specifically, Internet use, decision-making tools and monitoring systems. The proposal will focus on individuals with multi-morbidities, namely inflammatory arthritis plus on or more chronic conditions. This choice of study population is well justified.

Previous research shows that inequities may arise when people with chronic illness are unable to access health information on the internet. Those who were able to access such information identified positive impacts in support for self-management and pathways through care, learning from peers, gaining emotional support, and acquiring advice about treatment options. The question is whether a lack of access equates to a gap in healthcare and self-management support.

The significance of the study is well explained. The study will examine four categories of consumer e-health: internet use, peer-to-peer support or online forums, self- management applications, decision aids. It will also examine the very important issue of how access to e-health affects the patient-provider relationship and how physicians respond to e-health.

The study will explore an ethics framework for e-health using moral reasoning. While drawing on traditional bioethics principles of autonomy, beneficence and justice, the applicants will adopt a relational approach to understand the role and ramifications of e-health. This approach emphasizes context.

Assessment of the proposal

1. Research Approach

The applicants propose a series of 4-5 focus groups with patients and at least 2 with HCPs (recruitment strategies and inclusion criteria for patients and healthcare providers appropriate). These will be supplemented with interviews with approximately 12 patients and 12 HCPs, informed by themes emerging from focus groups.

The analyses will draw on aspects of grounded theory. The applicants will apply a ‘social constructionist version’ of grounded theory which aims to gain an interpretive understanding of social phenomena. They will attempt to construct theory from the data, and to draft an explanatory framework for future study. They will

Review Type/Type dévaluation: Committee Member 1/Membre de comité 1
Name of Applicant/Nom du chercheur: BACKMAN, Catherine Louise
Application No./Numéro de demande: 274771
Agency/Agence: CIHR/IRSC
Competition/Concours: 2012-03-01 Catalyst Grant: Ethics/Subvention catalyseur : éthique
Committee/Comité: Catalyst Grant: New Investigators and Mid-Career Investigators Transitioning into Ethics/Subvention catalyseur : Nouveaux chercheurs faisant la transition au domaine de l'éthique
Title/Titre: Exploring E-health ethics and multi-morbidity

Assessment/Évaluation:

draw on a narrative approach to hear people's storied accounts of their lives and experiences, how they build coherence, and link action with a moral purpose. Recognizing the moral themes of accounts fits with their focus on the ethics of healthcare and their overarching framework of relational ethics.

The applicants clearly describe their methodological approach. The Applicants acknowledge and address potential limitations.

2. *Originality*

This is a well-grounded and original piece of research on an important topic. It is based in prior research and with its new ethics focus, will lead to a more substantial application. The proposal is exceptionally well-written, with clear objectives, solid background research and justifications and a well-outlined methodology. While the issue of internet use for health information is not particularly novel, the focus on chronic diseases is. There is also value in unpacking the impact of e-health information on the physician-patient relationship

3. *Applicant(s)*

The applicants are well-positioned to carry out the research. **Catherine Backman** is an experienced health services researcher and Professor of Occupational Science and Occupational Therapy at UBC. Her work to date has focused on the impact of chronic illness and disability on participation in life roles. Backman's turn to ethical inquiry and relational ethics aligns with recurring themes in her research and teaching, and the core tenets of occupational therapy theory, practice, and the integral importance of social environment.

Anne Townsend is a junior colleague and medical sociologist and qualitative researcher at UBC who has been hired as a Research Associate by Catherine Backman. Her research addresses the social, ethical, clinical and health policy implications of chronic illness and emphasizes the moral dimensions of patient-provider relationships, self-management and consulting. Anne Townsend has been trained in health ethics.

Both Drs Backman and Townsend have demonstrated significant publication outputs relative to career stage. Their partnership has grown from a series of investigations on the impact of chronic illness on help-seeking and participation in everyday activity, where a number of ethical questions have arisen. The collaboration as it matures and continues has the potential to result in highly impactful research.

4. *Environment for the Research*

The proposal could have been strengthened by clarifying the role of Prof. Michael McDonald, a leading

**Review Type/Type dévaluation:**	Committee Member 1/Membre de comité 1
**Name of Applicant/Nom du chercheur:**	BACKMAN, Catherine Louise
**Application No./Numéro de demande:**	274771
**Agency/Agence:**	CIHR/IRSC
**Competition/Concours:**	2012-03-01 Catalyst Grant: Ethics/Subvention catalyseur : éthique
**Committee/Comité:**	Catalyst Grant: New Investigators and Mid-Career Investigators Transitioning into Ethics/Subvention catalyseur : Nouveaux chercheurs faisant la transition au domaine de l'éthique
**Title/Titre:**	Exploring E-health ethics and multi-morbidity

Assessment/Évaluation:

Canadian bioethicist and researcher at UBC. It is unlikely that adequate mentoring can be provided with a very minimal commitment from Prof. MacDonald.

5. Impact of the Research

The research will contribute to the currently poor understanding of the role of e-health in managing multi-morbidity from patient and HCP perspectives. E-health has been hailed as a catalyst for positive and sweeping improvements but there is only provisional empirical evidence on how consumers engage with e-health and conflicting evidence about its impact on patient-provider relationships. Focusing on experiences of multi-morbidity, to illuminate issues of use and need, this study will identify and analyze emerging ethical issues of e-health domains for self-management and patient provider relationships. The goal here is to inform a larger research project on this exceptionally important topic.

The applicants have outlined a knowledge translation strategy appropriate to the scope of project. Support from knowledge users and HCPs as collaborators and in letters add value to proposed research and enhance potential impact.

Budget

I would like to see the budget increased to support 2 full years of funding.

Review Type/Type dévaluation: Committee Member 2/Membre de comité 2
Name of Applicant/Nom du chercheur: BACKMAN, Catherine Louise
Application No./Numéro de demande: 274771
Agency/Agence: CIHR/IRSC
Competition/Concours: 2012-03-01 Catalyst Grant: Ethics/Subvention catalyseur : éthique
Committee/Comité: Catalyst Grant: New Investigators and Mid-Career Investigators Transitioning into Ethics/Subvention catalyseur : Nouveaux chercheurs faisant la transition au domaine de l'éthique
Title/Titre: Exploring E-health ethics and multi-morbidity

Assessment/Évaluation:

The proposal looks at the use of internet by persons with arthritis plus an additional condition to understand how they use the internet in the information-gathering, support, and self-management, how this affects their relationship with their providers, and identify the ethical issues involved in patient-driven internet use.

They have selected multi-ailment persons with arthritis because this is who they already work with, because of the prevalence of arthritis +, and because research on ehealth has primarily focused on single ailment.

Approach.

They are working within relational ethics; examining male-female differences; using focus groups and interviews. There are very standard procedures and may not get at anything particular unique about ehealth. It might have been more impressive if they looked at the novel technology of ehealth with methods more suited to understanding what “being on the internet” is like. There is work in folklore and phenomenology of this kind that has not been taken up here.

Originality.

This does not strike me as highly original. There is a lot of emerging work on multi-diagnosis, and while there is not a lot of ethics work in this area, the relational framework would seem to be the obvious way to go, so this doesn’t offer any special methodological or analytic value beyond what is already emerging.
Applicants.

Well-integrated team with considerable research expertise on the subject population. The PI is highly decorated as she makes her move into ethics. However, Townsend, the proposed ethics mentor, is not actually trained in ethics and does not have a substantial research record in that area. In addition, she has never served as a supervisor even for an MA student. This weakness in the ethics side likely explains the problems I identify in the approach

Impact

This is pretty weak. They argue that the value of ehealth may not be realized because it decreases autonomy and damages the patient-provider relationship. They say they will provide better contextual understanding,

**Review Type/Type dévaluation:**	Committee Member 2/Membre de comité 2
**Name of Applicant/Nom du chercheur:**	BACKMAN, Catherine Louise
**Application No./Numéro de demande:**	274771
**Agency/Agence:**	CIHR/IRSC
**Competition/Concours:**	2012-03-01 Catalyst Grant: Ethics/Subvention catalyseur : éthique
**Committee/Comité:**	Catalyst Grant: New Investigators and Mid-Career Investigators Transitioning into Ethics/Subvention catalyseur : Nouveaux chercheurs faisant la transition au domaine de l'éthique
**Title/Titre:**	Exploring E-health ethics and multi-morbidity

Assessment/Évaluation:

but there is not much novel thinking about how a relational framework improves ability to grapple with complexity of multi-ailment ehealth use.

Review Type/Type dévaluation: SO Notes /Notes de l'agent scientifique
Name of Applicant/Nom du chercheur: BACKMAN, Catherine Louise
Application No./Numéro de demande: 274771
Agency/Agence: CIHR/IRSC
Competition/Concours: 2012-03-01 Catalyst Grant: Ethics/Subvention catalyseur : éthique
Committee/Comité: Catalyst Grant: New Investigators and Mid-Career Investigators Transitioning into Ethics/Subvention catalyseur : Nouveaux chercheurs faisant la transition au domaine de l'éthique
Title/Titre: Exploring E-health ethics and multi-morbidity

Assessment/Évaluation:

Title: Exploring E-health ethics and multi-morbidity

Comments:

The committee was divided on this application. Members of the committee had a lengthy discussion about methods and approaches raised by this proposal. The distinction between sociology and ethics could be more clearly defined in this project to more effectively elicit the ethical component of this project and distinguish it from a more conventional sociological approach. For example, the concept of relational autonomy fits into both disciplines and further consideration as to how this explicitly engages with ethics and ethicists is needed. The ethics methodology is overly complex as presented and further reflection and refinement is encouraged to better clarify the ethical framework for this project. Members of the committee raised questions about the nature of mentorship in ethics specifically and the way in which ethics will be incorporated into the training model for the applicant.

Budget:

The committee had no specific recommendations on the budget, but the term should be extended to two years.
